# Supplementary material for: Global eye health and the sustainable development goals: protocol for a scoping review
Source: BMJ Open. 2020 Mar 17;10(3):e035789. doi: 10.1136/bmjopen-2019-035789 (PMC7202701; doi:10.1136/bmjopen-2019-035789)
Supplement: Supplementary data [file bmjopen-2019-035789supp001.pdf]

Appendix 1: Preferred Reporting Items for Systematic reviews and Meta-Analyses extension for Scoping Reviews (PRISMA-ScR) Checklist

SECTION	ITEM	PRISMA-ScR CHECKLIST ITEM	REPORTED ON PAGE #
**TITLE**			
Title	1	Identify the report as a scoping review.	1
**ABSTRACT**			
Structured summary	2	Provide a structured summary that includes (as applicable): background, objectives, eligibility criteria, sources of evidence, charting methods, results, and conclusions that relate to the review questions and objectives.	1
**INTRODUCTION**			
Rationale	3	Describe the rationale for the review in the context of what is already known. Explain why the review questions/objectives lend themselves to a scoping review approach.	1-2
Objectives	4	Provide an explicit statement of the questions and objectives being addressed with reference to their key elements (e.g., population or participants, concepts, and context) or other relevant key elements used to conceptualize the review questions and/or objectives.	2
**METHODS**			
Protocol and registration	5	Indicate whether a review protocol exists; state if and where it can be accessed (e.g., a Web address); and if available, provide registration information, including the registration number.	1, 2
Eligibility criteria	6	Specify characteristics of the sources of evidence used as eligibility criteria (e.g., years considered, language, and publication status), and provide a rationale.	2-3
Information sources	7	Describe all information sources in the search (e.g., databases with dates of coverage and contact with authors to identify additional sources), as well as the date the most recent search was executed.	3
Search	8	Present the full electronic search strategy for at least 1 database, including any limits used, such that it could be repeated.	3, Appendix 3
Selection of sources of evidence	9	State the process for selecting sources of evidence (i.e., screening and eligibility) included in the scoping review.	3
Data charting process	10	Describe the methods of charting data from the included sources of evidence (e.g., calibrated forms or forms that have been tested by the team before their use, and whether data charting was done independently or in duplicate) and any processes for obtaining and confirming data from investigators.	3
Data items	11	List and define all variables for which data were sought and any assumptions and simplifications made.	3
Critical appraisal of individual sources of evidence	12	If done, provide a rationale for conducting a critical appraisal of included sources of evidence; describe the methods used and how this information was used in any data synthesis (if appropriate).	N/A
Synthesis of results	13	Describe the methods of handling and summarizing the data that were charted.	3

From: Tricco AC, Lillie E, Zarin W, O'Brien KK, Colquhoun H, Levac D, et al. PRISMA Extension for Scoping Reviews (PRISMA-ScR): Checklist and Explanation. Ann Intern Med. ;169:467–473. doi: 10.7326/M18-0850

Appendix 2: Table of indicative SDG-related outcomes, and elements on the pathway between a change in eye health and an SDG.

SDG	SDG-related outcomes	Elements on the pathway
SDG 1: No poverty	• Poverty (all dimensions)<br>• Access to economic resources and microfinance initiatives	• Productivity<br>• Household per capita expenditure<br>• Social protection schemes<br>• Financial burden on, and sustainability of, the health system<br>• Efficient and effective use of resources<br>• Universal health coverage
SDG 2: Zero Hunger	• Nutrition<br>• Food security	• Vulnerability to malnutrition at the individual level<br>• Agricultural production
SDG 4: Quality education	• Educational outcomes such as literacy and numeracy rates<br>• Skills attainment for future employment<br>• Disparities in access to education / inclusive education	• School enrolment, attendance and retention
SDG 5: Gender equality	• Gender discrimination<br>• Violence against women and girls	• Gender equity in access to eye care services<br>• Empowerment of women and girls
SDG 6: Clean water and sanitation	• Access to safe, affordable drinking water<br>• Access to adequate sanitation and hygiene	• Strengthening of infrastructure for improved water, sanitation and hygiene
SDG 7: Affordable and clean energy	• Renewable energy<br>• Energy efficiency	
SDG 8: Decent work and economic growth	• Per capita economic growth<br>• Employment and decent work opportunities	• Worker productivity<br>• Presenteeism<br>• Absenteeism<br>• Retirement age
SDG 9: Industry, innovation and infrastructure	• Quality infrastructure<br>• Sustainable industrialisation<br>• Expansion of technology and innovation	
SDG 10: Reduced inequalities	• Social inclusion of all<br>• Economic inclusion of all<br>• Political inclusion of all	• Empowerment of vulnerable groups
SDG 11: Sustainable cities and communities	• Access to safe and affordable transport<br>• Access to safe and affordable housing<br>• Environmental impact of cities	• Road safety<br>• Environmental impact of the healthcare system, e.g. waste management
SDG 12: Responsible consumption and production		• Environmental impact of the healthcare system, e.g. waste management<br>• Recycling of eye care consumables
SDG 13: Climate action		
SDG 14: Life below water		
SDG 15: Life on land		
SDG 16: Peace, justice and strong institutions	• Levels of violence<br>• Levels of corruption	
SDG 17: Partnership	• Coalition-building partnerships	

Appendix 3: MEDLINE search terms

1. exp Ophthalmology/
2. exp Eye Diseases/
3. (trachoma$ or tracoma$ or trichiasis).tw.
4. (cataract$ or phaco$ or phako$).tw.
5. ((diabet$ or proliferat$) adj3 retinopath$).tw.
6. (amblyop$ or strabismus).tw.
7. exp Vision Tests/
8. Optometry/
9. (myopia or myopic or hyperop$ or hypermetrop$ or anisometrop$ or ammetrop$ or astigmati$ or presbyop$).tw.
10. (refractive adj1 error$).tw.
11. Eyeglasses/
12. (spectacle or spectacles or glasses).tw.
13. (eyeglasses or eye glasses).tw.
14. ((eye$ or vision or retina$ or ophthalm$ or retinopathy) adj2 exam$).tw.
15. ((eye$ or vision or retinopathy or ophthalm$) adj2 assess$).tw.
16. ((eye$ or vision or retina$ or ophthalm$ or retinopathy) adj2 test$).tw.
17. (eye$ adj2 (care or health or service$)).tw.
18. onchocerciasis.tw.
19. (river adj1 blindness).tw.
20. (retinopath$ adj2 prematur$).tw.
21. or/1-20
22. Trachoma/
23. (trachoma$ or tracoma$ or trichiasis).tw.
24. exp Cataract/
25. exp cataract Extraction/
26. cataract$.tw.
27. or/22-26
28. exp poverty/
29. poverty.tw.
30. poor people.tw.
31. (social adj2 protection).tw.
32. household expenditure.tw.
33. head of household.tw.
34. per capita expenditure.tw.
35. asset ownership.tw.
36. self-rated wealth.tw.
37. or/28-36
38. Malnutrition/
39. Malnutrition.tw.
40. (malnourish$ or undernourish$).tw.
41. (nutrition$ adj2 deficienc$).tw.
42. Vitamin A Deficiency/
43. Vitamin A deficien$.tw.
44. or/38-43
45. Education/
46. "Education of Visually Disabled"/
47. "Education of Intellectually Disabled"/
48. Remedial Teaching/
49. (school adj3 (performance or attain$ or achieve$ or attend$)).tw.
50. (educat$ adj2 (need$ or require$ or strateg$ or outcome$ or develop$)).tw.
51. (reading adj2 (attain$ or develop$)).tw.
52. (literacy or illiteracy or illiterate or numeracy).tw.
53. or/45-52
54. Women's Health/
55. Women's Health Services/
56. exp "sexual and gender minorities"/
57. (transgender or transsexual$).tw.
58. (gender adj1 identit$).tw.

Zhang JH, *et al. BMJ Open* 2020; 10:e035789. doi: 10.1136/bmjopen-2019-035789

59. (women$ adj1 health$).tw.
60. or/54-59
61. exp environmental health/
62. exp hygiene/
63. exp water supply/
64. ((wash$ or clean$ or hygien$) adj2 face$).tw.
65. ((wash$ or clean$ or hygien$) adj2 facial$).tw.
66. Insect Control/
67. exp insecticides/
68. or/61-67
69. Renewable Energy/
70. Energy-Generating Resources/
71. Power plants/
72. or/69-71
73. exp Employment/
74. Efficiency/
75. Efficiency, Organizational/
76. Sickness impact profile/
77. Occupational Diseases/
78. Absenteeism/
79. Presenteeism/
80. productivity.tw.
81. (product$ adj2 employ$).tw.
82. or/73-81
83. Organizational Innovation/
84. Sustainable Development/
85. or/83-84
86. Social Justice/
87. ((social$ or econom$ or politic$) adj3 inclu$).tw.
88. Healthcare Disparities/
89. Health Status Disparities/
90. (inequal$ or inequit$).tw.
91. equal opportun$.tw.
92. or/86-91
93. exp Motor Vehicles/
94. exp Automobile Driving/
95. exp Accidents, Traffic/
96. (road adj2 safe$).tw.
97. ((accident$ or crash$ or collision$) adj2 (road or car or vehicle$ or motor$ or traffic)).tw.
98. or/93-97
99. exp "Conservation of Natural Resources"/
100. exp Climate/
101. exp climate change/
102. (climate adj2 chang$).tw.
103. or/100-102
104. 37 or 44 or 53 or 60 or 72 or 82 or 85 or 92 or 98 or 99
105. 21 and 104
106. 68 or 103
107. 27 and 106
108. 105 or 107
109. exp animals/
110. exp humans/
111. 109 not (109 and 110)
112. 108 not 111
113. (animal$ or rabbit$ or rat or rats or mouse or mice or chicken$ or dog or dogs or cat or cats or feline or pig$ or monke$).tw.
114. 112 not 113
115. exp case reports/
116. (case$ adj3 (report$ or series)).tw.
117. 115 or 116
118. 114 not 117

119. (immunoblot$ or electron or mRNA or mitochondria$ or proteomic$ or metabolomic$ or allele or extracellular).tw.
120. (in vivo or in-vivo or in vitro or in-vitro).tw.
121. Urinary Tract Infections/
122. Gonorrhea/
123. Sexually Transmitted Diseases/
124. vagina/
125. pelvic inflammatory disease$.tw.
126. (gonorrhea$ or gonorrhoea$).tw.
127. (sexual$ adj3 transmit$).tw.
128. HIV.tw.
129. (ureth$ or urinary or genital$).tw.
130. (vagina$ or testes or testic$).tw.
131. visual analog$ scale$.tw.
132. (dental or oral$).ti.
133. or/119-132
134. 118 not 133

Zhang JH, *et al. BMJ Open* 2020; 10:e035789. doi: 10.1136/bmjopen-2019-035789
