# Supplementary material for: Global eye health and the sustainable development goals: protocol for a scoping review
Source: BMJ Open. 2020 Mar 17;10(3):e035789. doi: 10.1136/bmjopen-2019-035789 (PMC7202701; doi:10.1136/bmjopen-2019-035789)
Supplement: Author's manuscript [file bmjopen-2019-035789.draft_revisions.pdf]

BMJ Open is committed to open peer review. As part of this commitment we make the peer review history of every article we publish publicly available.

When an article is published we post the peer reviewers' comments and the authors' responses online. We also post the versions of the paper that were used during peer review. These are the versions that the peer review comments apply to.

The versions of the paper that follow are the versions that were submitted during the peer review process. They are not the versions of record or the final published versions. They should not be cited or distributed as the published version of this manuscript.

BMJ Open is an open access journal and the full, final, typeset and author-corrected version of record of the manuscript is available on our site with no access controls, subscription charges or pay-per-view fees (http://bmjopen.bmj.com).

If you have any questions on BMJ Open's open peer review process please email info.bmjopen@bmj.com

BMJ Open

Global Eye Health and the Sustainable Development Goals: Protocol for a Scoping Review

Journal:	*BMJ Open*
Manuscript ID	bmjopen-2019-035789
Article Type:	Protocol
Date Submitted by the Author:	15-Nov-2019

Complete List of Authors:	Zhang, Justine; London School of Hygiene and Tropical Medicine International Centre for Eye Health; Manchester Royal Eye Hospital<br>Ramke, Jacqueline; London School of Hygiene and Tropical Medicine International Centre for Eye Health; The University of Auckland, School of Optometry and Vision Science<br>Mwangi, Nyawira; London School of Hygiene and Tropical Medicine International Centre for Eye Health; Kenya Medical Training College, Department of Clinical Medicine<br>Furtado, João; Universidade de São Paulo Faculdade de Medicina de Ribeirão Preto, Division of Ophthalmology<br>Yasmin, Sumrana; Sight Savers International<br>Bascaran, Covadonga ; London School of Hygiene and Tropical Medicine International Centre for Eye Health<br>Ogundo, Cynthia; London School of Hygiene and Tropical Medicine International Centre for Eye Health; Mbagathi Hospital<br>Jan, Catherine; Peking University, School of Psychological and Cognitive Sciences<br>Gordon, Iris; London School of Hygiene and Tropical Medicine International Centre for Eye Health<br>Congdon, Nathan; Queen's University Belfast, Centre for Public Health<br>Burton, Matthew J; London School of Hygiene and Tropical Medicine International Centre for Eye Health; Moorfields Eye Hospital

Keywords:	OPHTHALMOLOGY, PUBLIC HEALTH, International health services < HEALTH SERVICES ADMINISTRATION & MANAGEMENT

SCHOLARONE™
Manuscripts

BMJ

I, the Submitting Author has the right to grant and does grant on behalf of all authors of the Work (as defined in the below author licence), an exclusive licence and/or a non-exclusive licence for contributions from authors who are: i) UK Crown employees; ii) where BMJ has agreed a CC-BY licence shall apply, and/or iii) in accordance with the terms applicable for US Federal Government officers or employees acting as part of their official duties; on a worldwide, perpetual, irrevocable, royalty-free basis to BMJ Publishing Group Ltd ("BMJ") its licensees and where the relevant Journal is co-owned by BMJ to the co-owners of the Journal, to publish the Work in this journal and any other BMJ products and to exploit all rights, as set out in our licence.

The Submitting Author accepts and understands that any supply made under these terms is made by BMJ to the Submitting Author unless you are acting as an employee on behalf of your employer or a postgraduate student of an affiliated institution which is paying any applicable article publishing charge ("APC") for Open Access articles. Where the Submitting Author wishes to make the Work available on an Open Access basis (and intends to pay the relevant APC), the terms of reuse of such Open Access shall be governed by a Creative Commons licence – details of these licences and which Creative Commons licence will apply to this Work are set out in our licence referred to above.

Other than as permitted in any relevant BMJ Author's Self Archiving Policies, I confirm this Work has not been accepted for publication elsewhere, is not being considered for publication elsewhere and does not duplicate material already published. I confirm all authors consent to publication of this Work and authorise the granting of this licence.

For peer review only

GLOBAL EYE HEALTH AND THE SUSTAINABLE DEVELOPMENT GOALS: PROTOCOL FOR A SCOPING REVIEW

Authors:

Justine H. Zhang[1, 2], Jacqueline Ramke[1, 3], Nyawira Mwangi[1, 4], João Furtado[5], Sumrana Yasmin[6], Covadonga Bascaran[1], Cynthia Ogundo[1, 7], Catherine Jan[8], Iris Gordon[1], Nathan Congdon[9], Matthew J. Burton[1, 10]

Affiliations:

1. International Centre for Eye Health, London School of Hygiene and Tropical Medicine, London, United Kingdom
2. Manchester Royal Eye Hospital, Manchester, United Kingdom
3. School of Optometry and Vision Science, University of Auckland, New Zealand
4. Department of Clinical Medicine, Kenya Medical Training College, Nairobi, Kenya
5. Division of Ophthalmology, Ribeirão Preto Medical School, University of São Paulo, Ribeirão Preto, São Paulo, Brazil
6. Sightsavers, Islamabad, Pakistan
7. Mbagathi Hospital, Nairobi, Kenya
8. School of Psychological and Cognitive Sciences, Peking University, Beijing, China
9. Centre for Public Health, Queen's University, Belfast, United Kingdom
10. Moorfields Eye Hospital, London, United Kingdom

For peer review only

For peer review only - http://bmjopen.bmj.com/site/about/guidelines.xhtml

ABSTRACT

Introduction

In 2015, most governments of the world committed to achieving 17 Sustainable Development Goals (SDGs) by the year 2030. Efforts to improve eye health contribute to the advancement of several SDGs, including those not exclusively health-related. This scoping review summarises the nature and extent of the published literature that demonstrates a link between improved eye health and advancement of the SDGs.

Methods and analysis

Searches will be conducted in MEDLINE, Embase and Global Health for published, peer-reviewed manuscripts, with no time period, language, or geographic limits. All intervention and observational studies will be included if they report a link between a change in eye health and (1) an outcome related to one of the SDGs, or (2) an element on a pathway between eye health and an SDG (e.g. productivity).

Two investigators will independently screen titles and abstracts, followed by full-text screening of potentially relevant articles. Reference lists of all included articles will be examined to identify further potentially relevant studies. Conflicts between the two independent investigators will be discussed and resolved with a third investigator. For included articles, data regarding publication characteristics, study details, and SDG-related outcomes will be extracted. Results will be synthesised by mapping the extracted data to a logic model, which will be refined through an iterative process during data synthesis.

Ethics and dissemination

As this scoping review will only include published data, ethics approval will not be sought. The findings of the review will be published in an open-access, peer-reviewed journal. A summary of the results will be developed for website posting, stakeholder meetings, and inclusion in the ongoing Lancet Global Health Commission on Global Eye Health.

Registration details: https://osf.io/gu4z6/ (DOI 10.17605/OSF.IO/GU4Z6)

For peer review only

For peer review only - http://bmjopen.bmj.com/site/about/guidelines.xhtml

STRENGTHS AND LIMITATIONS OF THIS STUDY

- This is the first review to determine the nature and extent of published literature on how improvements in eye health contribute to the advancement of the SDGs.
- The review will comprehensively assess published peer-reviewed manuscripts, with no time period, language, or geographic restrictions.
- A potential limitation might be the paucity of published literature on how eye health contributes to some of the SDGs.
- Another potential limitation is that the complexity of pathways between eye health and the SDGs is unlikely to be fully appreciated from the published literature.

For peer review only

For peer review only - http://bmjopen.bmj.com/site/about/guidelines.xhtml

INTRODUCTION

In 2015, at the United Nations (UN) Sustainable Development Summit most governments committed to the Sustainable Development Goals (SDGs). The 17 SDGs have 169 targets and 232 indicators that UN member states aim to achieve by 2030.[1] These goals are broad and far-reaching, with a vision for global change that encompasses the advancement of economic, health, education, equality, social, and environmental issues.

We hypothesise that improved eye health contributes to the advancement of multiple SDGs. The aim of this review is to summarise the nature and extent of the published literature that demonstrates this link between improved eye health and the advancement of the SDGs. We consider improved eye health to include the full range of promotion, prevention, treatment, and rehabilitation strategies.[2] Improving eye health is not only about improving sight, but also, reducing disability, morbidity (e.g. pain) and improving wellbeing. We chose to undertake a scoping review rather than an alternative evidence synthesis approach because we wished to identify and map the available evidence, which we anticipate will be heterogeneous.[3]

To guide our review, we developed a model to conceptualise how improved eye health leads to realisation of a range of SDGs (Figure 1).[4] To develop this model we asked Commissioners of the ongoing Lancet Global Health Commission on Global Eye Health to review each of the 169 SDG targets and outline the links (hypothesized or established) between any of these targets and eye health.[1, 5] These links were reviewed, and an initial logic model drafted and iteratively refined with input by the authorship group.[4] In the model (Figure 1), we have depicted SDG 3 as improved eye health. The SDGs relating to the environment, peace and partnership are cross-cutting themes that should be borne in mind at every step of the pathway.

We recognise that eye health has consequences for other health and wellbeing outcomes, but in this scoping review we aim to identify the broader societal implications linked to eye health. Alongside this review, and also within the Lancet Global Health Commission on Global Eye Health, we are undertaking a complementary review of the intersections between eye health and other health and wellbeing outcomes that will be published separately.

We also recognise that some of the relationships between eye health and the SDGs might be bi-directional. For example, reduced hunger (SDG 2) reduces malnutrition-related eye disease (and thus improves eye health), but conversely, improved eye health reduces poverty and thus reduces hunger (SDG 2). However, for the purposes of this review, we are focused on the contribution that improved eye health can make to other SDGs.

For peer review only - http://bmjopen.bmj.com/site/about/guidelines.xhtml

For peer review only

Objectives / Scoping review questions

We aim to answer the following questions:

1. What is the nature and extent of the published evidence that improving eye health contributes to advancement of the SDGs?
2. What are the main pathways by which improving eye health leads to advancement of the SDGs?

For peer review only

For peer review only - http://bmjopen.bmj.com/site/about/guidelines.xhtml

METHODS AND ANALYSIS

Protocol and registration

This protocol is reported according to the relevant sections of the PRISMA Extension for Scoping Reviews (PRISMA-ScR) Checklist (Appendix 1). The protocol has been registered prospectively with Open Science Framework: https://osf.io/gu4z6/ (DOI 10.17605/OSF.IO/GU4Z6).

Eligibility criteria

Type of study: All intervention and observational studies will be included if they report a link between a change in eye health and: (1) an outcome related to one of the SDGs (see Appendix 2 for list of indicative outcomes), or (2) an element on a pathway between eye health and an SDG (e.g. productivity; see Appendix 2 for list of indicative pathway elements).

Time period: All time periods.

Setting: Studies can be from any world region.

Language: Manuscripts in all languages will be included for screening. Best efforts will be made to translate any foreign language publications. For any publication where translation is not possible, we will refer to English language versions of their abstracts (if available).

Publication status: Published peer-reviewed manuscripts only. As this scoping review is concerned with identifying the extent of evidence in published literature, grey literature will not be searched.

Search

We will search MEDLINE, Embase and Global Health using a search strategy developed by an Information Specialist from Cochrane Eyes and Vision (IG) (MEDLINE search strategy included in Appendix 3). We will examine reference lists of all included articles to identify further potentially relevant studies. Following the selection process, field experts will be provided a list of the included studies and requested to identify further potentially relevant studies.

Selection of sources of evidence

All titles and abstracts will be screened by two investigators independently using Covidence systematic review software (Veritas Health Innovation, Melbourne, Australia. Available at www.covidence.org). Subsequently, full texts will be assessed by two investigators independently to establish eligibility for inclusion into the study, and reasons for exclusion will be assigned by each reviewer. Any conflicts will be discussed and resolved with a third reviewer. A PRISMA flow diagram will be completed to summarise the study selection process.

Data charting process

Data charting forms will be developed in Excel and tested by all investigators prior to use. Data charting of included studies will be performed by two investigators independently. We anticipate a broad scope of included studies, so data charting will be an iterative process throughout the review and the data charting

For peer review only

form will be amended as required. We plan to contact study authors in the case of unclear information and will make up to three attempts by email.

Data items

- Publication characteristics:
    - Authors
    - Date of publication
    - Journal
    - Country of study
    - Source of funding and role of funder
    - Type of study: (1a) Intervention, randomised; (1b) Intervention, non-randomised; (1c) Intervention, model; (2a) Observational, cohort, analytical; (2b) Observational, cohort, descriptive; (2c) Observational, case-control; (2d) Observational, cross-sectional, analytical; (2e) Observational, cross-sectional, descriptive; (2f) Observational, ecological; (3) Other – please state
- Study details:
    - Exposure(s)/intervention(s)
    - Outcome(s)
    - Effect estimate(s)
- SDG-related characteristics:
    - Identify relevant SDG (or multiple relevant SDGs)
    - Specify outcome(s) related to one of more of the SDGs (and targets if possible)
    - Describe the link between eye health and the SDG(s) (and the pathway if available)
    - Map to logic model (Figure 1)
    - Identify any links with the environment SDGs (SDG 7, 12, 13, 14 and 15)
    - Identify any links with peace and partnership SDGs (SDG 16 and 17)

Synthesis of results

Following data charting, results will be synthesised by mapping the retrieved evidence to an Eye Health - SDG logic model. The model in Figure 1 will be used as a dynamic tool to aid this synthesis, and refined through an iterative process. Each pathway on the Eye Health - SDG logic model will then be examined separately and relevant evidence for that pathway will be collated and summarised, including measures of effectiveness where available. The extent of evidence supporting each pathway on the logic model will be indicated, e.g. by differing text types.[6] If sufficient homogenous studies are found on a particular SDG-related outcome we will consider meta-analysis, and will develop a detailed protocol for this separately.

Patient and Public Involvement Statement

As we plan to review existing published literature only, this scoping review will be performed without specific patient involvement.

For peer review only - http://bmjopen.bmj.com/site/about/guidelines.xhtml
For peer review only

ETHICS AND DISSEMINATION

Ethics approval is not required, as our review will only include published data. We will publish our findings in an open-access, peer-reviewed journal and develop an accessible summary of the results for website posting and stakeholder meetings. A summary of the results will also be included in the ongoing Lancet Global Health Commission on Global Eye Health.[5] We anticipate that the findings will be of considerable interest to those involved in Eye Health provision as useful information for advocacy and to the wider development community.

For peer review only

REFERENCES

1. United Nations General Assembly. Transforming our World: The 2030 Agenda for Sustainable Development. Resolution Adopted by the General Assembly on 25 September 2015. New York, NY, USA: United Nations General Assembly, 2015.
2. World Health Organisation. World Report on Vision. Geneva: World Health Organisation, 2019.
3. Munn Z, Peters MDJ, Stern C, et al. Systematic review or scoping review? Guidance for authors when choosing between a systematic or scoping review approach. *BMC Med Res Methodol* 2018;18(1):143. doi: 10.1186/s12874-018-0611-x [published Online First: 2018/11/21]
4. Kneale D, Thomas J, Harris K. Developing and Optimising the Use of Logic Models in Systematic Reviews: Exploring Practice and Good Practice in the Use of Programme Theory in Reviews. *PLoS One* 2015;10(11):e0142187. doi: 10.1371/journal.pone.0142187 [published Online First: 2015/11/18]
5. Burton MJ, Faal HB, Ramke J, et al. Announcing The Lancet Global Health Commission on Global Eye Health. *Lancet Glob Health* 2019 doi: 10.1016/S2214-109X(19)30450-4 [published Online First: 2019/10/14]
6. Baxter SK, Blank L, Woods HB, et al. Using logic model methods in systematic review synthesis: describing complex pathways in referral management interventions. *BMC Med Res Methodol* 2014;14:62. doi: 10.1186/1471-2288-14-62 [published Online First: 2014/06/03]
7. United Nations. Sustainable Development Goals: Communications materials. [Available from: https://www.un.org/sustainabledevelopment/news/communications-material/ accessed 11/09/2019.

For peer review only

For peer review only - http://bmjopen.bmj.com/site/about/guidelines.xhtml

Authors' Contributions: MJB conceived the idea for the review. JHZ, JR, MJB and NC developed the logic model. JHZ and JR drafted and revised the protocol with suggestions from MJB, NC, NM, CB, SY, JF, CO, CJ who reviewed the protocol and provided feedback on the draft. IG constructed the search.

Corresponding Author: Dr Justine Zhang (justine.zhang@lshtm.ac.uk)

Funding Statement: MJB is supported by the Wellcome Trust (207472/Z/17/Z). JR is a Commonwealth Rutherford Fellow, funded by the UK government through the Commonwealth Scholarship Commission in the UK. The Lancet Global Health Commission on Global Eye Health is supported by The Queen Elizabeth Diamond Jubilee Trust, The Wellcome Trust, Sightsavers, The Fred Hollows Foundation, The SEVA Foundation, The British Council for the Prevention of Blindness and Christian Blind Mission.

For peer review only

Acknowledgements: We acknowledge colleagues and Commissioners of the Lancet Global Health Commission on Global Eye Health who provided suggested links between eye health and the SDGs during the model development process (Brandon Ah Tong, Andrew Bastawrous, John Buchan, Rènée du Toit, Hannah Faal, Michael Gichangi, Clare Gilbert, Jost Jonas, Hannah Kuper, Fatima Kyari, Van Charles Lansingh, Ana Patrícia Marques, Ciku Mathenge, GVS Murthy, Thulasiraj Ravilla, Juan Carlos Silva, Anthony Solomon, Bonnielin Swenor, Hugh Taylor, Aubrey Webson).

Competing Interests: None declared.

Number of Tables: 0

Number of Figures: 1

Data Sharing Statement: Data generated from this review will be available upon reasonable request from Dr Justine Zhang (justine.zhang@lshtm.ac.uk)

Keywords: eye health, eye care, ophthalmology, health, Sustainable Development Goals

Word Count: 1372

Legends

Figure 1: Logic model outlining examples of pathways by which improved eye health may contribute to the achievement of the Sustainable Development Goals.[7] (Arrows represent directionality, and not necessarily causality; the links between eye health and 'SDG 3 Good Health and Well-being' are considered in a complimentary review).

For peer review only

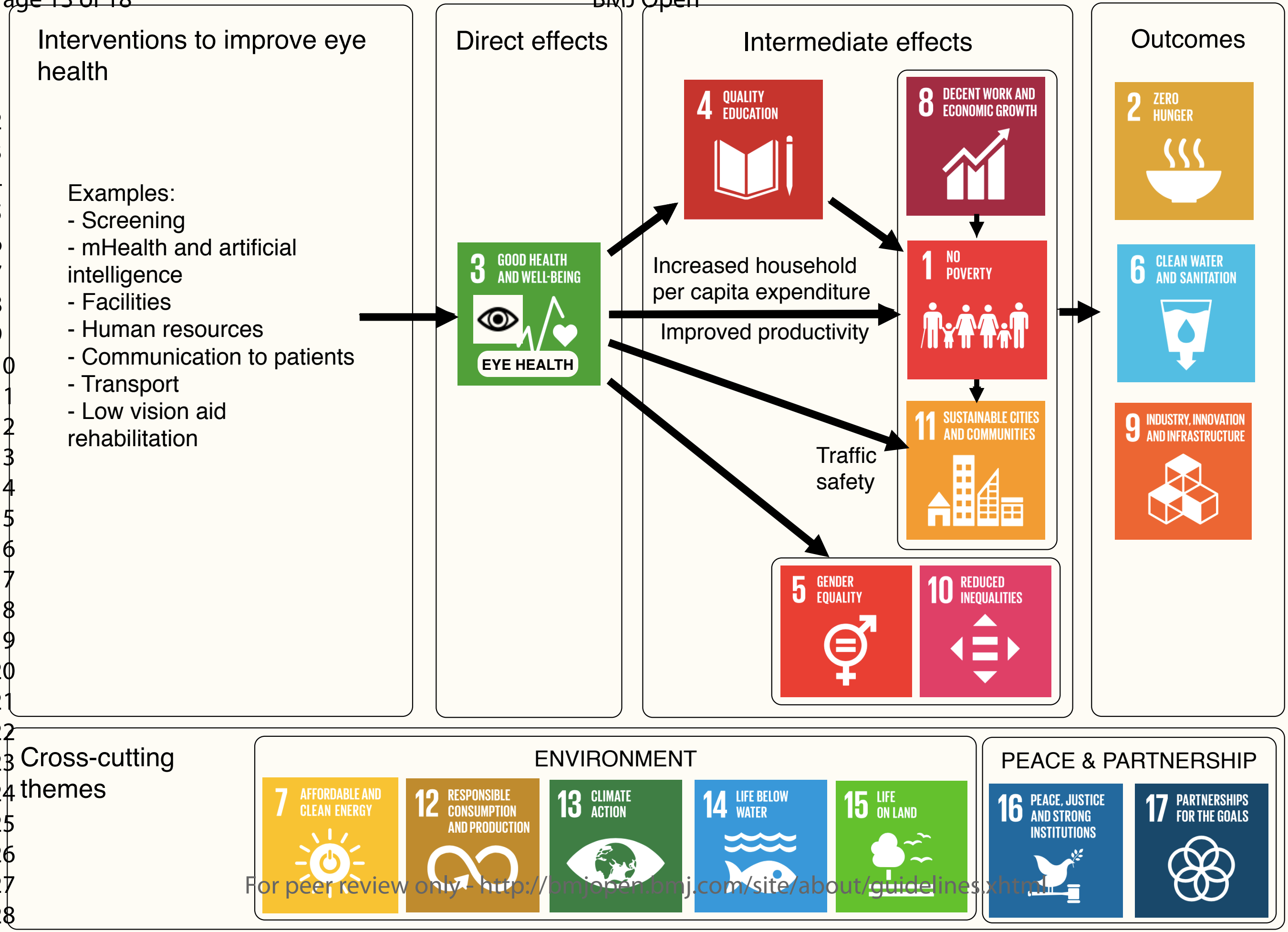

For peer review only - http://bmjopen.bmj.com/site/about/guidelines.xhtml

Appendix 1: Preferred Reporting Items for Systematic reviews and Meta-Analyses extension for Scoping Reviews (PRISMA-ScR) Checklist

SECTION	ITEM	PRISMA-ScR CHECKLIST ITEM	REPORTED ON PAGE #
**TITLE**			
Title	1	Identify the report as a scoping review.	1
**ABSTRACT**			
Structured summary	2	Provide a structured summary that includes (as applicable): background, objectives, eligibility criteria, sources of evidence, charting methods, results, and conclusions that relate to the review questions and objectives.	2
**INTRODUCTION**			
Rationale	3	Describe the rationale for the review in the context of what is already known. Explain why the review questions/objectives lend themselves to a scoping review approach.	4-5
Objectives	4	Provide an explicit statement of the questions and objectives being addressed with reference to their key elements (e.g., population or participants, concepts, and context) or other relevant key elements used to conceptualize the review questions and/or objectives.	5
**METHODS**			
Protocol and registration	5	Indicate whether a review protocol exists; state if and where it can be accessed (e.g., a Web address); and if available, provide registration information, including the registration number.	2, 6
Eligibility criteria	6	Specify characteristics of the sources of evidence used as eligibility criteria (e.g., years considered, language, and publication status), and provide a rationale.	6
Information sources	7	Describe all information sources in the search (e.g., databases with dates of coverage and contact with authors to identify additional sources), as well as the date the most recent search was executed.	6
Search	8	Present the full electronic search strategy for at least 1 database, including any limits used, such that it could be repeated.	6, Appendix 3
Selection of sources of evidence	9	State the process for selecting sources of evidence (i.e., screening and eligibility) included in the scoping review.	6
Data charting process	10	Describe the methods of charting data from the included sources of evidence (e.g., calibrated forms or forms that have been tested by the team before their use, and whether data charting was done independently or in duplicate) and any processes for obtaining and confirming data from investigators.	6-7
Data items	11	List and define all variables for which data were sought and any assumptions and simplifications made.	7
Critical appraisal of individual sources of evidence	12	If done, provide a rationale for conducting a critical appraisal of included sources of evidence; describe the methods used and how this information was used in any data synthesis (if appropriate).	N/A
Synthesis of results	13	Describe the methods of handling and summarizing the data that were charted.	7

From: Tricco AC, Lillie E, Zarin W, O'Brien KK, Colquhoun H, Levac D, et al. PRISMA Extension for Scoping Reviews (PRISMA-ScR): Checklist and Explanation. Ann Intern Med. ;169:467–473. doi: 10.7326/M18-0850

For peer review only

Appendix 2: Table of indicative SDG-related outcomes, and elements on the pathway between a change in eye health and an SDG.

SDG	SDG-related outcomes	Elements on the pathway
SDG 1: No poverty	• Poverty (all dimensions)<br>• Access to economic resources and microfinance initiatives	• Productivity<br>• Household per capita expenditure<br>• Social protection schemes<br>• Financial burden on, and sustainability of, the health system<br>• Efficient and effective use of resources<br>• Universal health coverage
SDG 2: Zero Hunger	• Nutrition<br>• Food security	• Vulnerability to malnutrition at the individual level<br>• Agricultural production
SDG 4: Quality education	• Educational outcomes such as literacy and numeracy rates<br>• Skills attainment for future employment<br>• Disparities in access to education / inclusive education	• School enrolment, attendance and retention
SDG 5: Gender equality	• Gender discrimination<br>• Violence against women and girls	• Gender equity in access to eye care services<br>• Empowerment of women and girls
SDG 6: Clean water and sanitation	• Access to safe, affordable drinking water<br>• Access to adequate sanitation and hygiene	• Strengthening of infrastructure for improved water, sanitation and hygiene
SDG 7: Affordable and clean energy	• Renewable energy<br>• Energy efficiency	
SDG 8: Decent work and economic growth	• Per capita economic growth<br>• Employment and decent work opportunities	• Worker productivity<br>• Presenteeism<br>• Absenteeism<br>• Retirement age
SDG 9: Industry, innovation and infrastructure	• Quality infrastructure<br>• Sustainable industrialisation<br>• Expansion of technology and innovation	
SDG 10: Reduced inequalities	• Social inclusion of all<br>• Economic inclusion of all<br>• Political inclusion of all	• Empowerment of vulnerable groups
SDG 11: Sustainable cities and communities	• Access to safe and affordable transport<br>• Access to safe and affordable housing<br>• Environmental impact of cities	• Road safety<br>• Environmental impact of the healthcare system, e.g. waste management
SDG 12: Responsible consumption and production		• Environmental impact of the healthcare system, e.g. waste management<br>• Recycling of eye care consumables
SDG 13: Climate action		
SDG 14: Life below water		
SDG 15: Life on land		
SDG 16: Peace, justice and strong institutions	• Levels of violence<br>• Levels of corruption	
SDG 17: Partnership	• Coalition-building partnerships	

For peer review only

For peer review only - http://bmjopen.bmj.com/site/about/guidelines.xhtml

Appendix 3: MEDLINE search terms

1. exp Ophthalmology/
2. exp Eye Diseases/
3. (trachoma$ or tracoma$ or trichiasis).tw.
4. (cataract$ or phaco$ or phako$).tw.
5. ((diabet$ or proliferat$) adj3 retinopath$).tw.
6. (amblyop$ or strabismus).tw.
7. exp Vision Tests/
8. Optometry/
9. (myopia or myopic or hyperop$ or hypermetrop$ or anisometrop$ or ammetrop$ or astigmati$ or presbyop$).tw.
10. (refractive adj1 error$).tw.
11. Eyeglasses/
12. (spectacle or spectacles or glasses).tw.
13. (eyeglasses or eye glasses).tw.
14. ((eye$ or vision or retina$ or ophthalm$ or retinopathy) adj2 exam$).tw.
15. ((eye$ or vision or retinopathy or ophthalm$) adj2 assess$).tw.
16. ((eye$ or vision or retina$ or ophthalm$ or retinopathy) adj2 test$).tw.
17. (eye$ adj2 (care or health or service$)).tw.
18. onchocerciasis.tw.
19. (river adj1 blindness).tw.
20. (retinopath$ adj2 prematur$).tw.
21. or/1-20
22. Trachoma/
23. (trachoma$ or tracoma$ or trichiasis).tw.
24. exp Cataract/
25. exp cataract Extraction/
26. cataract$.tw.
27. or/22-26
28. exp poverty/
29. poverty.tw.
30. poor people.tw.
31. (social adj2 protection).tw.
32. household expenditure.tw.
33. head of household.tw.
34. per capita expenditure.tw.
35. asset ownership.tw.
36. self-rated wealth.tw.
37. or/28-36
38. Malnutrition/
39. Malnutrition.tw.
40. (malnourish$ or undernourish$).tw.
41. (nutrition$ adj2 deficienc$).tw.
42. Vitamin A Deficiency/
43. Vitamin A deficien$.tw.
44. or/38-43
45. Education/
46. "Education of Visually Disabled"/
47. "Education of Intellectually Disabled"/
48. Remedial Teaching/
49. (school adj3 (performance or attain$ or achieve$ or attend$)).tw.
50. (educat$ adj2 (need$ or require$ or strateg$ or outcome$ or develop$)).tw.
51. (reading adj2 (attain$ or develop$)).tw.
52. (literacy or illiteracy or illiterate or numeracy).tw.
53. or/45-52
54. Women's Health/
55. Women's Health Services/
56. exp "sexual and gender minorities"/
57. (transgender or transsexual$).tw.
58. (gender adj1 identit$).tw.

For peer review only

59. (women$ adj1 health$).tw.
60. or/54-59
61. exp environmental health/
62. exp hygiene/
63. exp water supply/
64. ((wash$ or clean$ or hygien$) adj2 face$).tw.
65. ((wash$ or clean$ or hygien$) adj2 facial$).tw.
66. Insect Control/
67. exp insecticides/
68. or/61-67
69. Renewable Energy/
70. Energy-Generating Resources/
71. Power plants/
72. or/69-71
73. exp Employment/
74. Efficiency/
75. Efficiency, Organizational/
76. Sickness impact profile/
77. Occupational Diseases/
78. Absenteeism/
79. Presenteeism/
80. productivity.tw.
81. (product$ adj2 employ$).tw.
82. or/73-81
83. Organizational Innovation/
84. Sustainable Development/
85. or/83-84
86. Social Justice/
87. ((social$ or econom$ or politic$) adj3 inclu$).tw.
88. Healthcare Disparities/
89. Health Status Disparities/
90. (inequal$ or inequit$).tw.
91. equal opportun$.tw.
92. or/86-91
93. exp Motor Vehicles/
94. exp Automobile Driving/
95. exp Accidents, Traffic/
96. (road adj2 safe$).tw.
97. ((accident$ or crash$ or collision$) adj2 (road or car or vehicle$ or motor$ or traffic)).tw.
98. or/93-97
99. exp "Conservation of Natural Resources"/
100. exp Climate/
101. exp climate change/
102. (climate adj2 chang$).tw.
103. or/100-102
104. 37 or 44 or 53 or 60 or 72 or 82 or 85 or 92 or 98 or 99
105. 21 and 104
106. 68 or 103
107. 27 and 106
108. 105 or 107
109. exp animals/
110. exp humans/
111. 109 not (109 and 110)
112. 108 not 111
113. (animal$ or rabbit$ or rat or rats or mouse or mice or chicken$ or dog or dogs or cat or cats or feline or pig$ or monke$).tw.
114. 112 not 113
115. exp case reports/
116. (case$ adj3 (report$ or series)).tw.
117. 115 or 116
118. 114 not 117

For peer review only

119. (immunoblot$ or electron or mRNA or mitochondria$ or proteomic$ or metabolomic$ or allele or extracellular).tw.
120. (in vivo or in-vivo or in vitro or in-vitro).tw.
121. Urinary Tract Infections/
122. Gonorrhea/
123. Sexually Transmitted Diseases/
124. vagina/
125. pelvic inflammatory disease$.tw.
126. (gonorrhea$ or gonorrhoea$).tw.
127. (sexual$ adj3 transmit$).tw.
128. HIV.tw.
129. (ureth$ or urinary or genital$).tw.
130. (vagina$ or testes or testic$).tw.
131. visual analog$ scale$.tw.
132. (dental or oral$).ti.
133. or/119-132
134. 118 not 133

For peer review only

For peer review only - http://bmjopen.bmj.com/site/about/guidelines.xhtml

Preferred Reporting Items for Systematic reviews and Meta-Analyses extension for Scoping Reviews (PRISMA-ScR) Checklist

SECTION	ITEM	PRISMA-ScR CHECKLIST ITEM	REPORTED ON PAGE #
**TITLE**			
Title	1	Identify the report as a scoping review.	1
**ABSTRACT**			
Structured summary	2	Provide a structured summary that includes (as applicable): background, objectives, eligibility criteria, sources of evidence, charting methods, results, and conclusions that relate to the review questions and objectives.	2
**INTRODUCTION**			
Rationale	3	Describe the rationale for the review in the context of what is already known. Explain why the review questions/objectives lend themselves to a scoping review approach.	4-5
Objectives	4	Provide an explicit statement of the questions and objectives being addressed with reference to their key elements (e.g., population or participants, concepts, and context) or other relevant key elements used to conceptualize the review questions and/or objectives.	5
**METHODS**			
Protocol and registration	5	Indicate whether a review protocol exists; state if and where it can be accessed (e.g., a Web address); and if available, provide registration information, including the registration number.	2, 6
Eligibility criteria	6	Specify characteristics of the sources of evidence used as eligibility criteria (e.g., years considered, language, and publication status), and provide a rationale.	6
Information sources	7	Describe all information sources in the search (e.g., databases with dates of coverage and contact with authors to identify additional sources), as well as the date the most recent search was executed.	6
Search	8	Present the full electronic search strategy for at least 1 database, including any limits used, such that it could be repeated.	6, Appendix 3
Selection of sources of evidence	9	State the process for selecting sources of evidence (i.e., screening and eligibility) included in the scoping review.	6
Data charting process	10	Describe the methods of charting data from the included sources of evidence (e.g., calibrated forms or forms that have been tested by the team before their use, and whether data charting was done independently or in duplicate) and any processes for obtaining and confirming data from investigators.	6-7
Data items	11	List and define all variables for which data were sought and any assumptions and simplifications made.	7
Critical appraisal of individual sources of evidence	12	If done, provide a rationale for conducting a critical appraisal of included sources of evidence; describe the methods used and how this information was used in any data synthesis (if appropriate).	N/A
Synthesis of results	13	Describe the methods of handling and summarizing the data that were charted.	7

From: Tricco AC, Lillie E, Zarin W, O'Brien KK, Colquhoun H, Levac D, et al. PRISMA Extension for Scoping Reviews (PRISMA-ScR): Checklist and Explanation. Ann Intern Med. ;169:467–473. doi: 10.7326/M18-0850

For peer review only

BMJ Open

Global Eye Health and the Sustainable Development Goals: Protocol for a Scoping Review

Journal:	*BMJ Open*
Manuscript ID	bmjopen-2019-035789.R1
Article Type:	Protocol
Date Submitted by the Author:	13-Jan-2020
Complete List of Authors:	Zhang, Justine; London School of Hygiene and Tropical Medicine International Centre for Eye Health; Manchester Royal Eye Hospital<br>Ramke, Jacqueline; London School of Hygiene and Tropical Medicine International Centre for Eye Health; The University of Auckland, School of Optometry and Vision Science<br>Mwangi, Nyawira; London School of Hygiene and Tropical Medicine International Centre for Eye Health; Kenya Medical Training College, Department of Clinical Medicine<br>Furtado, João; Universidade de São Paulo Faculdade de Medicina de Ribeirão Preto, Division of Ophthalmology<br>Yasmin, Sumrana; Sight Savers International<br>Bascaran, Covadonga ; London School of Hygiene and Tropical Medicine International Centre for Eye Health<br>Ogundo, Cynthia; London School of Hygiene and Tropical Medicine International Centre for Eye Health; Mbagathi Hospital<br>Jan, Catherine; Peking University, School of Psychological and Cognitive Sciences<br>Gordon, Iris; London School of Hygiene and Tropical Medicine International Centre for Eye Health<br>Congdon, Nathan; Queen's University Belfast, Centre for Public Health<br>Burton, Matthew J; London School of Hygiene and Tropical Medicine International Centre for Eye Health; Moorfields Eye Hospital
**Primary Subject Heading**:	Ophthalmology
Secondary Subject Heading:	Global health, Public health, Health services research
Keywords:	OPHTHALMOLOGY, PUBLIC HEALTH, International health services < HEALTH SERVICES ADMINISTRATION & MANAGEMENT

SCHOLARONE™ Manuscripts

I, the Submitting Author has the right to grant and does grant on behalf of all authors of the Work (as defined in the below author licence), an exclusive licence and/or a non-exclusive licence for contributions from authors who are: i) UK Crown employees; ii) where BMJ has agreed a CC-BY licence shall apply, and/or iii) in accordance with the terms applicable for US Federal Government officers or employees acting as part of their official duties; on a worldwide, perpetual, irrevocable, royalty-free basis to BMJ Publishing Group Ltd ("BMJ") its licensees and where the relevant Journal is co-owned by BMJ to the co-owners of the Journal, to publish the Work in this journal and any other BMJ products and to exploit all rights, as set out in our licence.

The Submitting Author accepts and understands that any supply made under these terms is made by BMJ to the Submitting Author unless you are acting as an employee on behalf of your employer or a postgraduate student of an affiliated institution which is paying any applicable article publishing charge ("APC") for Open Access articles. Where the Submitting Author wishes to make the Work available on an Open Access basis (and intends to pay the relevant APC), the terms of reuse of such Open Access shall be governed by a Creative Commons licence – details of these licences and which Creative Commons licence will apply to this Work are set out in our licence referred to above.

Other than as permitted in any relevant BMJ Author's Self Archiving Policies, I confirm this Work has not been accepted for publication elsewhere, is not being considered for publication elsewhere and does not duplicate material already published. I confirm all authors consent to publication of this Work and authorise the granting of this licence.

For peer review only

GLOBAL EYE HEALTH AND THE SUSTAINABLE DEVELOPMENT GOALS: PROTOCOL FOR A SCOPING REVIEW

Authors:

Justine H. Zhang[1, 2], Jacqueline Ramke[1, 3], Nyawira Mwangi[1, 4], João Furtado[5], Sumrana Yasmin[6], Covadonga Bascaran[1], Cynthia Ogundo[1, 7], Catherine Jan[8], Iris Gordon[1], Nathan Congdon[9], Matthew J. Burton[1, 10]

Affiliations:

1. International Centre for Eye Health, London School of Hygiene and Tropical Medicine, London, United Kingdom
2. Manchester Royal Eye Hospital, Manchester, United Kingdom
3. School of Optometry and Vision Science, University of Auckland, New Zealand
4. Department of Clinical Medicine, Kenya Medical Training College, Nairobi, Kenya
5. Division of Ophthalmology, Ribeirão Preto Medical School, University of São Paulo, Ribeirão Preto, São Paulo, Brazil
6. Sightsavers, Islamabad, Pakistan
7. Mbagathi Hospital, Nairobi, Kenya
8. School of Psychological and Cognitive Sciences, Peking University, Beijing, China
9. Centre for Public Health, Queen's University, Belfast, United Kingdom
10. Moorfields Eye Hospital, London, United Kingdom

Corresponding Author: Dr Justine Zhang (justine.zhang@lshtm.ac.uk)

review only

For peer review only - http://bmjopen.bmj.com/site/about/guidelines.xhtml

ABSTRACT

Introduction

In 2015, most governments of the world committed to achieving 17 Sustainable Development Goals (SDGs) by the year 2030. Efforts to improve eye health contribute to the advancement of several SDGs, including those not exclusively health-related. This scoping review will summarise the nature and extent of the published literature that demonstrates a link between improved eye health and advancement of the SDGs.

Methods and analysis

Searches will be conducted in MEDLINE, Embase and Global Health for published, peer-reviewed manuscripts, with no time period, language, or geographic limits. All intervention and observational studies will be included if they report a link between a change in eye health and (1) an outcome related to one of the SDGs, or (2) an element on a pathway between eye health and an SDG (e.g. productivity).

Two investigators will independently screen titles and abstracts, followed by full-text screening of potentially relevant articles. Reference lists of all included articles will be examined to identify further potentially relevant studies. Conflicts between the two independent investigators will be discussed and resolved with a third investigator. For included articles, data regarding publication characteristics, study details, and SDG-related outcomes will be extracted. Results will be synthesised by mapping the extracted data to a logic model, which will be refined through an iterative process during data synthesis.

Ethics and dissemination

As this scoping review will only include published data, ethics approval will not be sought. The findings of the review will be published in an open-access, peer-reviewed journal. A summary of the results will be developed for website posting, stakeholder meetings, and inclusion in the ongoing Lancet Global Health Commission on Global Eye Health.

Registration details: https://osf.io/gu4z6/ (DOI 10.17605/OSF.IO/GU4Z6)

For peer review only

For peer review only - http://bmjopen.bmj.com/site/about/guidelines.xhtml

STRENGTHS AND LIMITATIONS OF THIS STUDY

- This is the first review to determine the nature and extent of published literature on how improvements in eye health contribute to the advancement of the SDGs.
- The review will comprehensively assess published peer-reviewed manuscripts, with no time period, language, or geographic restrictions.
- A potential limitation might be the paucity of published literature on how eye health contributes to some of the SDGs.
- Another potential limitation is that the complexity of pathways between eye health and the SDGs is unlikely to be fully appreciated from the published literature.

For peer review only

INTRODUCTION

In 2015, at the United Nations (UN) Sustainable Development Summit most governments around the world committed to the Sustainable Development Goals (SDGs). The 17 SDGs have 169 targets and 232 indicators that UN member states aim to achieve by 2030.[1] The SDGs build on the Millennium Development Goals and UN member states are expected to use the SDGs as a framework to guide the development of national and international policies. The SDGs are broad and far-reaching, with a vision for global change that encompasses the advancement of economic, health, education, equality, social, and environmental issues. All of the SDGs are interlinked, for example, improving health goes hand-in-hand with ending poverty, reduction of inequalities, and strengthening the economy.

We hypothesise that improved eye health contributes to the advancement of multiple SDGs. The aim of this review is to summarise the nature and extent of the published literature that demonstrates this link between improved eye health and the advancement of the SDGs. We consider improved eye health to include the full range of promotion, prevention, treatment, and rehabilitation strategies.[2] Improving eye health is not only about improving sight, but also, reducing disability, morbidity (e.g. pain) and improving wellbeing. We chose to undertake a scoping review rather than an alternative evidence synthesis approach because we wished to identify and map the available evidence, which we anticipate will be heterogeneous.[3]

To guide our review, we developed a model to conceptualise how improved eye health leads to realisation of a range of SDGs (Figure 1).[4] To develop this model we asked Commissioners of the ongoing Lancet Global Health Commission on Global Eye Health to review each of the 169 SDG targets and outline the links (hypothesised or established) between any of these targets and eye health.[1, 5] These links were reviewed, and an initial logic model drafted and iteratively refined with input by the authorship group.[4] In the model (Figure 1), we have depicted SDG 3 as improved eye health, since eye health comes under the umbrella of 'SDG 3 Health and Wellbeing'. The SDGs relating to the environment, peace and partnership are cross-cutting themes that should be borne in mind at every step of the pathway.

We recognise that eye health has consequences for other health and wellbeing outcomes, but in this scoping review we aim to identify the broader societal implications linked to eye health. Alongside this review, and also within the Lancet Global Health Commission on Global Eye Health, we are undertaking a complementary review of the intersections between eye health and other health and wellbeing outcomes that will be published separately.

We also recognise that some of the relationships between eye health and the SDGs might be bi-directional. For example, reduced hunger (SDG 2) reduces malnutrition-related eye disease (and thus improves eye health), but conversely, improved eye health reduces poverty and thus reduces hunger (SDG 2). However, for the purposes of this review, we are focused on the contribution that improved eye health can make to other SDGs.

For peer review only - http://bmjopen.bmj.com/site/about/guidelines.xhtml

Objectives / Scoping review questions

We aim to answer the following questions:

1. What is the nature and extent of the published evidence that improving eye health contributes to advancement of the SDGs?
2. What are the main pathways by which improving eye health leads to advancement of the SDGs?

For peer review only

METHODS AND ANALYSIS

Protocol and registration

This protocol is reported according to the relevant sections of the PRISMA Extension for Scoping Reviews (PRISMA-ScR) Checklist (Appendix 1). The protocol has been registered prospectively with Open Science Framework: https://osf.io/gu4z6/ (DOI 10.17605/OSF.IO/GU4Z6).

Eligibility criteria

Type of study: Primary research studies or meta-analyses only. All intervention and observational studies will be included if they report a link between a change in eye health and: (1) an outcome related to one of the SDGs (see Appendix 2 for list of indicative outcomes), or (2) an element on a pathway between eye health and an SDG (e.g. productivity; see Appendix 2 for list of indicative pathway elements). For systematic reviews without a meta-analysis, we will examine the reference lists of the systematic review for potentially relevant studies but the actual systematic review will not be included.

Time period: All time periods.

Setting: Studies can be from any world region.

Language: Manuscripts in all languages will be included for screening. Best efforts will be made to translate any foreign language publications. For any publication where translation is not possible, we will refer to English language versions of their abstract (if available).

Publication status: Published peer-reviewed primary research studies or meta-analyses only. As this scoping review is concerned with identifying the extent of evidence in published literature, grey literature will not be searched.

Search

We will search MEDLINE, Embase and Global Health using a search strategy developed by an Information Specialist from Cochrane Eyes and Vision (IG). The search was constructed using a set of terms describing eye health, and they were combined with a set of terms describing each of the SDGs (except for SDG 3). Our search excluded the following study designs by using 'stop words': animal, laboratory, case reports, and case series. Our MEDLINE search strategy is included in Appendix 3. We will download and de-duplicate the results in EndNote, and then export the results in to Covidence for screening. We will examine reference lists of all included articles to identify further potentially relevant studies. Following the selection process, field experts will be provided a list of the included studies and requested to identify further potentially relevant studies.

Selection of sources of evidence

All titles and abstracts will be screened by two investigators independently using Covidence systematic review software (Veritas Health Innovation, Melbourne, Australia. Available at www.covidence.org). Subsequently, full texts will be assessed by two investigators independently to establish eligibility for inclusion into the study, and reasons for exclusion will be assigned by each reviewer. Any conflicts will be discussed

For peer review only - http://bmjopen.bmj.com/site/about/guidelines.xhtml

and resolved with a third reviewer. A PRISMA flow diagram will be completed to summarise the study selection process.

Data charting process

Data charting forms will be developed in Excel and tested by all investigators on two studies each prior to use, based on the data items listed below. Data charting of included studies will be performed by two investigators independently. We anticipate a broad scope of included studies, so data charting will be an iterative process throughout the review and the data charting form will be amended as required. We plan to contact study authors in the case of unclear information and will make up to three attempts by email.

Data items

- Publication characteristics:
    - Authors
    - Date of publication
    - Journal
    - Country of study
    - Source of funding and role of funder
    - Type of study: (1a) Intervention, randomised; (1b) Intervention, non-randomised; (1c) Intervention, model; (2a) Observational, cohort, analytical; (2b) Observational, cohort, descriptive; (2c) Observational, case-control; (2d) Observational, cross-sectional, analytical; (2e) Observational, cross-sectional, descriptive; (2f) Observational, ecological; (3) Other – please state
- Study details:
    - Exposure(s)/intervention(s)
    - Outcome(s)
    - Effect estimate(s)
- SDG-related characteristics:
    - Identify relevant SDG (or multiple relevant SDGs)
    - Specify outcome(s) related to one or more of the SDGs (and targets if possible)
    - Describe the link between eye health and the SDG(s) (and the pathway if available)
    - Map to logic model (Figure 1)
    - Identify any links with the environment SDGs (SDG 7, 12, 13, 14 and 15)
    - Identify any links with peace and partnership SDGs (SDG 16 and 17)

Synthesis of results

Following data charting, results will be synthesised by mapping the retrieved evidence to an Eye Health - SDG logic model. The model in Figure 1 will be used as a dynamic tool to aid this synthesis, and refined through an iterative process. Each pathway on the Eye Health - SDG logic model will then be examined separately and relevant evidence for that pathway will be collated and summarised, including

For peer review only - http://bmjopen.bmj.com/site/about/guidelines.xhtml

measures of effectiveness where available. The extent of evidence supporting each pathway on the logic model will be indicated, e.g. by differing font styles or typographical emphasis (e.g. italics).[6] If sufficient homogenous studies are found on a particular SDG-related outcome we will consider meta-analysis, and will develop a detailed protocol for this separately.

Patient and Public Involvement Statement

This protocol was developed with input from the Commissioners of the Lancet Global Health Commission on Global Eye Health, which includes a diverse internationally representative group that includes people with lived experience of vision impairment, policy makers, academics, clinicians, government eye health programme leaders and advocacy specialists (please see Acknowledgements section for full list of involved Commissioners).

ETHICS & DISSEMINATION

Ethics approval is not required for this review, as it will only include published data. The results of this review will be used to develop a framework for how eye health contributes to the advancement of the SDGs. We will publish our findings in an open-access, peer-reviewed journal and develop an accessible summary of the results for website posting and stakeholder meetings. A summary of the results will also be included in the ongoing Lancet Global Health Commission on Global Eye Health.[5] We anticipate that the findings of this work will be of considerable interest to multiple stakeholders: people with lived experience of vision impairment, eye health professionals, clinicians, policy makers, and the development community. We expect the information will be useful for policy debate and advocacy in the wider development community. It will also guide eye health researchers to where there are gaps in evidence and identify areas for future research.

For peer review only

For peer review only - http://bmjopen.bmj.com/site/about/guidelines.xhtml

REFERENCES

1. United Nations General Assembly. Transforming our World: The 2030 Agenda for Sustainable Development. Resolution Adopted by the General Assembly on 25 September 2015. New York, NY, USA: United Nations General Assembly, 2015.
2. World Health Organisation. World Report on Vision. Geneva: World Health Organisation, 2019.
3. Munn Z, Peters MDJ, Stern C, et al. Systematic review or scoping review? Guidance for authors when choosing between a systematic or scoping review approach. *BMC Med Res Methodol* 2018;18(1):143. doi: 10.1186/s12874-018-0611-x [published Online First: 2018/11/21]
4. Kneale D, Thomas J, Harris K. Developing and Optimising the Use of Logic Models in Systematic Reviews: Exploring Practice and Good Practice in the Use of Programme Theory in Reviews. *PLoS One* 2015;10(11):e0142187. doi: 10.1371/journal.pone.0142187 [published Online First: 2015/11/18]
5. Burton MJ, Faal HB, Ramke J, et al. Announcing The Lancet Global Health Commission on Global Eye Health. *Lancet Glob Health* 2019 doi: 10.1016/S2214-109X(19)30450-4 [published Online First: 2019/10/14]
6. Baxter SK, Blank L, Woods HB, et al. Using logic model methods in systematic review synthesis: describing complex pathways in referral management interventions. *BMC Med Res Methodol* 2014;14:62. doi: 10.1186/1471-2288-14-62 [published Online First: 2014/06/03]
7. United Nations. Sustainable Development Goals: Communications materials. [Available from: https://www.un.org/sustainabledevelopment/news/communications-material/ accessed 11/09/2019.

For peer review only

Authors' Contributions: MJB conceived the idea for the review. JHZ, JR, MJB and NC developed the logic model. JHZ and JR drafted and revised the protocol with suggestions from MJB, NC, NM, CB, SY, JF, CO, CJ who reviewed the protocol and provided feedback on the draft. IG constructed the search.

Funding Statement: MJB is supported by the Wellcome Trust (207472/Z/17/Z). JR is a Commonwealth Rutherford Fellow, funded by the UK government through the Commonwealth Scholarship Commission in the UK. The Lancet Global Health Commission on Global Eye Health is supported by The Queen Elizabeth Diamond Jubilee Trust, Moorfields Eye Charity (grant number GR001061), NIHR Moorfields Biomedical Research Centre, The Wellcome Trust, Sightsavers, The Fred Hollows Foundation, The SEVA Foundation, The British Council for the Prevention of Blindness and Christian Blind Mission.

Acknowledgements: We acknowledge colleagues and Commissioners of the Lancet Global Health Commission on Global Eye Health who provided suggested links between eye health and the SDGs during the model development process (Brandon Ah Tong, Andrew Bastawrous, John Buchan, Rènée du Toit, Hannah Faal, Michael Gichangi, Clare Gilbert, Jost Jonas, Hannah Kuper, Fatima Kyari, Van Charles Lansingh, Ana Patrícia Marques, Ciku Mathenge, GVS Murthy, Thulasiraj Ravilla, Juan Carlos Silva, Anthony Solomon, Bonnielin Swenor, Hugh Taylor, Aubrey Webson).

Competing Interests: None declared.

Number of Tables: 0

Number of Figures: 1

Data Sharing Statement: Data generated from this review will be available upon reasonable request from Dr Justine Zhang (justine.zhang@lshtm.ac.uk)

Keywords: eye health, eye care, ophthalmology, health, Sustainable Development Goals

Word Count: 1670

For peer review only

Legends

Figure 1: Logic model outlining examples of pathways by which improved eye health may contribute to the achievement of the Sustainable Development Goals.[7] (Arrows represent directionality, and not necessarily causality; the links between eye health and 'SDG 3 Good Health and Well-being' are considered in a complimentary review).

For peer review only

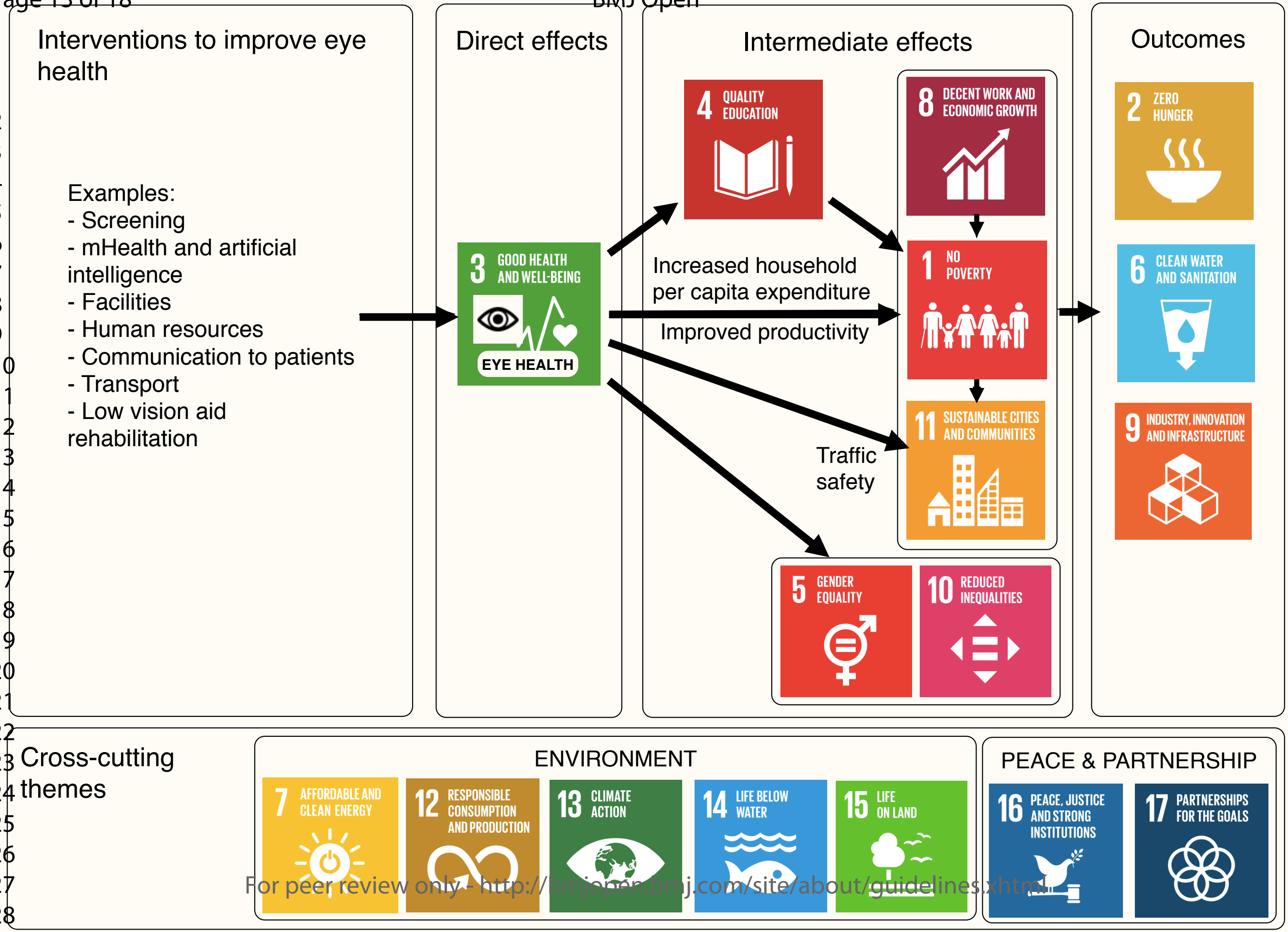

For peer review only - http://bmjopen.bmj.com/site/about/guidelines.xhtml

Appendix 1: Preferred Reporting Items for Systematic reviews and Meta-Analyses extension for Scoping Reviews (PRISMA-ScR) Checklist

SECTION	ITEM	PRISMA-ScR CHECKLIST ITEM	REPORTED ON PAGE #
**TITLE**			
Title	1	Identify the report as a scoping review.	1
**ABSTRACT**			
Structured summary	2	Provide a structured summary that includes (as applicable): background, objectives, eligibility criteria, sources of evidence, charting methods, results, and conclusions that relate to the review questions and objectives.	2
**INTRODUCTION**			
Rationale	3	Describe the rationale for the review in the context of what is already known. Explain why the review questions/objectives lend themselves to a scoping review approach.	4-5
Objectives	4	Provide an explicit statement of the questions and objectives being addressed with reference to their key elements (e.g., population or participants, concepts, and context) or other relevant key elements used to conceptualize the review questions and/or objectives.	5
**METHODS**			
Protocol and registration	5	Indicate whether a review protocol exists; state if and where it can be accessed (e.g., a Web address); and if available, provide registration information, including the registration number.	2, 6
Eligibility criteria	6	Specify characteristics of the sources of evidence used as eligibility criteria (e.g., years considered, language, and publication status), and provide a rationale.	6
Information sources	7	Describe all information sources in the search (e.g., databases with dates of coverage and contact with authors to identify additional sources), as well as the date the most recent search was executed.	6
Search	8	Present the full electronic search strategy for at least 1 database, including any limits used, such that it could be repeated.	6, Appendix 3
Selection of sources of evidence	9	State the process for selecting sources of evidence (i.e., screening and eligibility) included in the scoping review.	6
Data charting process	10	Describe the methods of charting data from the included sources of evidence (e.g., calibrated forms or forms that have been tested by the team before their use, and whether data charting was done independently or in duplicate) and any processes for obtaining and confirming data from investigators.	7
Data items	11	List and define all variables for which data were sought and any assumptions and simplifications made.	7
Critical appraisal of individual sources of evidence	12	If done, provide a rationale for conducting a critical appraisal of included sources of evidence; describe the methods used and how this information was used in any data synthesis (if appropriate).	N/A
Synthesis of results	13	Describe the methods of handling and summarizing the data that were charted.	7-8

From: Tricco AC, Lillie E, Zarin W, O'Brien KK, Colquhoun H, Levac D, et al. PRISMA Extension for Scoping Reviews (PRISMA-ScR): Checklist and Explanation. Ann Intern Med. ;169:467–473. doi: 10.7326/M18-0850

For peer review only - http://bmjopen.bmj.com/site/about/guidelines.xhtml

Appendix 2: Table of indicative SDG-related outcomes, and elements on the pathway between a change in eye health and an SDG.

SDG	SDG-related outcomes	Elements on the pathway
SDG 1: No poverty	• Poverty (all dimensions)<br>• Access to economic resources and microfinance initiatives	• Productivity<br>• Household per capita expenditure<br>• Social protection schemes<br>• Financial burden on, and sustainability of, the health system<br>• Efficient and effective use of resources<br>• Universal health coverage
SDG 2: Zero Hunger	• Nutrition<br>• Food security	• Vulnerability to malnutrition at the individual level<br>• Agricultural production
SDG 4: Quality education	• Educational outcomes such as literacy and numeracy rates<br>• Skills attainment for future employment<br>• Disparities in access to education / inclusive education	• School enrolment, attendance and retention
SDG 5: Gender equality	• Gender discrimination<br>• Violence against women and girls	• Gender equity in access to eye care services<br>• Empowerment of women and girls
SDG 6: Clean water and sanitation	• Access to safe, affordable drinking water<br>• Access to adequate sanitation and hygiene	• Strengthening of infrastructure for improved water, sanitation and hygiene
SDG 7: Affordable and clean energy	• Renewable energy<br>• Energy efficiency	
SDG 8: Decent work and economic growth	• Per capita economic growth<br>• Employment and decent work opportunities	• Worker productivity<br>• Presenteeism<br>• Absenteeism<br>• Retirement age
SDG 9: Industry, innovation and infrastructure	• Quality infrastructure<br>• Sustainable industrialisation<br>• Expansion of technology and innovation	
SDG 10: Reduced inequalities	• Social inclusion of all<br>• Economic inclusion of all<br>• Political inclusion of all	• Empowerment of vulnerable groups
SDG 11: Sustainable cities and communities	• Access to safe and affordable transport<br>• Access to safe and affordable housing<br>• Environmental impact of cities	• Road safety<br>• Environmental impact of the healthcare system, e.g. waste management
SDG 12: Responsible consumption and production		• Environmental impact of the healthcare system, e.g. waste management<br>• Recycling of eye care consumables
SDG 13: Climate action		
SDG 14: Life below water		
SDG 15: Life on land		
SDG 16: Peace, justice and strong institutions	• Levels of violence<br>• Levels of corruption	
SDG 17: Partnership	• Coalition-building partnerships	

For peer review only

For peer review only - http://bmjopen.bmj.com/site/about/guidelines.xhtml

Appendix 3: MEDLINE search terms

1. exp Ophthalmology/
2. exp Eye Diseases/
3. (trachoma$ or tracoma$ or trichiasis).tw.
4. (cataract$ or phaco$ or phako$).tw.
5. ((diabet$ or proliferat$) adj3 retinopath$).tw.
6. (amblyop$ or strabismus).tw.
7. exp Vision Tests/
8. Optometry/
9. (myopia or myopic or hyperop$ or hypermetrop$ or anisometrop$ or ammetrop$ or astigmati$ or presbyop$).tw.
10. (refractive adj1 error$).tw.
11. Eyeglasses/
12. (spectacle or spectacles or glasses).tw.
13. (eyeglasses or eye glasses).tw.
14. ((eye$ or vision or retina$ or ophthalm$ or retinopathy) adj2 exam$).tw.
15. ((eye$ or vision or retinopathy or ophthalm$) adj2 assess$).tw.
16. ((eye$ or vision or retina$ or ophthalm$ or retinopathy) adj2 test$).tw.
17. (eye$ adj2 (care or health or service$)).tw.
18. onchocerciasis.tw.
19. (river adj1 blindness).tw.
20. (retinopath$ adj2 prematur$).tw.
21. or/1-20
22. Trachoma/
23. (trachoma$ or tracoma$ or trichiasis).tw.
24. exp Cataract/
25. exp cataract Extraction/
26. cataract$.tw.
27. or/22-26
28. exp poverty/
29. poverty.tw.
30. poor people.tw.
31. (social adj2 protection).tw.
32. household expenditure.tw.
33. head of household.tw.
34. per capita expenditure.tw.
35. asset ownership.tw.
36. self-rated wealth.tw.
37. or/28-36
38. Malnutrition/
39. Malnutrition.tw.
40. (malnourish$ or undernourish$).tw.
41. (nutrition$ adj2 deficienc$).tw.
42. Vitamin A Deficiency/
43. Vitamin A deficien$.tw.
44. or/38-43
45. Education/
46. "Education of Visually Disabled"/
47. "Education of Intellectually Disabled"/
48. Remedial Teaching/
49. (school adj3 (performance or attain$ or achieve$ or attend$)).tw.
50. (educat$ adj2 (need$ or require$ or strateg$ or outcome$ or develop$)).tw.
51. (reading adj2 (attain$ or develop$)).tw.
52. (literacy or illiteracy or illiterate or numeracy).tw.
53. or/45-52
54. Women's Health/
55. Women's Health Services/
56. exp "sexual and gender minorities"/
57. (transgender or transsexual$).tw.
58. (gender adj1 identit$).tw.

For peer review only

For peer review only - http://bmjopen.bmj.com/site/about/guidelines.xhtml

59. (women$ adj1 health$).tw.
60. or/54-59
61. exp environmental health/
62. exp hygiene/
63. exp water supply/
64. ((wash$ or clean$ or hygien$) adj2 face$).tw.
65. ((wash$ or clean$ or hygien$) adj2 facial$).tw.
66. Insect Control/
67. exp insecticides/
68. or/61-67
69. Renewable Energy/
70. Energy-Generating Resources/
71. Power plants/
72. or/69-71
73. exp Employment/
74. Efficiency/
75. Efficiency, Organizational/
76. Sickness impact profile/
77. Occupational Diseases/
78. Absenteeism/
79. Presenteeism/
80. productivity.tw.
81. (product$ adj2 employ$).tw.
82. or/73-81
83. Organizational Innovation/
84. Sustainable Development/
85. or/83-84
86. Social Justice/
87. ((social$ or econom$ or politic$) adj3 inclu$).tw.
88. Healthcare Disparities/
89. Health Status Disparities/
90. (inequal$ or inequit$).tw.
91. equal opportun$.tw.
92. or/86-91
93. exp Motor Vehicles/
94. exp Automobile Driving/
95. exp Accidents, Traffic/
96. (road adj2 safe$).tw.
97. ((accident$ or crash$ or collision$) adj2 (road or car or vehicle$ or motor$ or traffic)).tw.
98. or/93-97
99. exp "Conservation of Natural Resources"/
100. exp Climate/
101. exp climate change/
102. (climate adj2 chang$).tw.
103. or/100-102
104. 37 or 44 or 53 or 60 or 72 or 82 or 85 or 92 or 98 or 99
105. 21 and 104
106. 68 or 103
107. 27 and 106
108. 105 or 107
109. exp animals/
110. exp humans/
111. 109 not (109 and 110)
112. 108 not 111
113. (animal$ or rabbit$ or rat or rats or mouse or mice or chicken$ or dog or dogs or cat or cats or feline or pig$ or monke$).tw.
114. 112 not 113
115. exp case reports/
116. (case$ adj3 (report$ or series)).tw.
117. 115 or 116
118. 114 not 117

For peer review only - http://bmjopen.bmj.com/site/about/guidelines.xhtml

119. (immunoblot$ or electron or mRNA or mitochondria$ or proteomic$ or metabolomic$ or allele or extracellular).tw.
120. (in vivo or in-vivo or in vitro or in-vitro).tw.
121. Urinary Tract Infections/
122. Gonorrhea/
123. Sexually Transmitted Diseases/
124. vagina/
125. pelvic inflammatory disease$.tw.
126. (gonorrhea$ or gonorrhoea$).tw.
127. (sexual$ adj3 transmit$).tw.
128. HIV.tw.
129. (ureth$ or urinary or genital$).tw.
130. (vagina$ or testes or testic$).tw.
131. visual analog$ scale$.tw.
132. (dental or oral$).ti.
133. or/119-132
134. 118 not 133

For peer review only

Preferred Reporting Items for Systematic reviews and Meta-Analyses extension for Scoping Reviews (PRISMA-ScR) Checklist

SECTION	ITEM	PRISMA-ScR CHECKLIST ITEM	REPORTED ON PAGE #
**TITLE**			
Title	1	Identify the report as a scoping review.	1
**ABSTRACT**			
Structured summary	2	Provide a structured summary that includes (as applicable): background, objectives, eligibility criteria, sources of evidence, charting methods, results, and conclusions that relate to the review questions and objectives.	2
**INTRODUCTION**			
Rationale	3	Describe the rationale for the review in the context of what is already known. Explain why the review questions/objectives lend themselves to a scoping review approach.	4-5
Objectives	4	Provide an explicit statement of the questions and objectives being addressed with reference to their key elements (e.g., population or participants, concepts, and context) or other relevant key elements used to conceptualize the review questions and/or objectives.	5
**METHODS**			
Protocol and registration	5	Indicate whether a review protocol exists; state if and where it can be accessed (e.g., a Web address); and if available, provide registration information, including the registration number.	2, 6
Eligibility criteria	6	Specify characteristics of the sources of evidence used as eligibility criteria (e.g., years considered, language, and publication status), and provide a rationale.	6
Information sources	7	Describe all information sources in the search (e.g., databases with dates of coverage and contact with authors to identify additional sources), as well as the date the most recent search was executed.	6
Search	8	Present the full electronic search strategy for at least 1 database, including any limits used, such that it could be repeated.	6, Appendix 3
Selection of sources of evidence	9	State the process for selecting sources of evidence (i.e., screening and eligibility) included in the scoping review.	6
Data charting process	10	Describe the methods of charting data from the included sources of evidence (e.g., calibrated forms or forms that have been tested by the team before their use, and whether data charting was done independently or in duplicate) and any processes for obtaining and confirming data from investigators.	7
Data items	11	List and define all variables for which data were sought and any assumptions and simplifications made.	7
Critical appraisal of individual sources of evidence	12	If done, provide a rationale for conducting a critical appraisal of included sources of evidence; describe the methods used and how this information was used in any data synthesis (if appropriate).	N/A
Synthesis of results	13	Describe the methods of handling and summarizing the data that were charted.	7-8

From: Tricco AC, Lillie E, Zarin W, O'Brien KK, Colquhoun H, Levac D, et al. PRISMA Extension for Scoping Reviews (PRISMA-ScR): Checklist and Explanation. Ann Intern Med. ;169:467–473. doi: 10.7326/M18-0850

For peer review only

For peer review only - http://bmjopen.bmj.com/site/about/guidelines.xhtml
